# Commentary: Probabilistic Representation in Human Visual Cortex Reflects Uncertainty in Serial Decisions

**DOI:** 10.3389/fnhum.2020.580581

**Published:** 2020-10-21

**Authors:** Raymundo Machado De Azevedo Neto

**Affiliations:** Instituto do Cérebro, Hospital Israelita Albert Einstein, São Paulo, Brazil

**Keywords:** serial dependence, uncertainty, probabilistic population code, Bayesian ideal observer, short-term memory (STM)

Humans combine incoming sensory and past information to deal with ambiguous and uncertain information (e.g., Knill and Saunders, 2003). But what constitutes past information? Many studies have recently reported that the last presented stimulus exerts an attractive bias at perceptual reports for many visual stimulus features (e.g., Fischer and Whitney, 2014). This phenomenon has been termed serial dependence, and has been cast as a consequence of optimal weighting of current and previous stimulus information based on their uncertainty (Cicchini et al., 2018).

But how does the brain represent uncertainty associated with a particular percept? Bayesian theories of neural coding propose that a population of neurons represent each stimulus as a probability distribution and that the uncertainty associated with stimulus processing is encoded as the width of this probability distribution (see Pouget et al., 2013 for a review). In addition, these theories propose that the observer uses such uncertainty in the decision-making process.

If serial dependence relies on the combination of current and past sensory information weighted by their uncertainty, then stimulus uncertainty encoded at the sensory processing stage should affect the serial dependence effect. Response bias toward the last seen stimulus should be greater when current stimulus uncertainty is higher than that of the previously seen stimulus. In a recent paper, van Bergen and Jehee (2019) tested this hypothesis in an elegant combination of psychophysical experiment, fMRI acquisition and computational modeling. They had participants perform a delayed reproduction task, where they briefly showed participants oriented gratings in sequence, and, after a short delay between each stimulus, asked them to reproduce the orientation of the remembered grating on a computer screen. As expected, van Bergen and Jehee (2019) replicated the serial dependence effect by showing that the reproduced orientation was biased toward the orientation of the previously seen grating.

To test the prediction that sensory uncertainty encoded in the population activity of visual areas modulate the serial dependence effect, the authors decoded the trial-by-trial uncertainty of population activity using BOLD fMRI (van Bergen et al., 2015). They used a multivoxel pattern analysis to fit a probabilistic model to a subset of the data where the activation pattern across voxels in the visual cortex (V1, V2, and V3) is associated with a particular stimulus orientation. Then, the authors estimated the probability distribution of possible grating orientations on an independent subset of the data, given the pattern of activation across voxels and the previously fitted parameters. The resulting posterior probability distribution provides information about the most probable stimulus presented on a given trial and uncertainty associated with the represented stimulus. This uncertainty, represented by the width of the probability distribution, is assumed to be a measure of the degree of uncertainty present in the population activation pattern.

The authors grouped two sets of trials according to previous and current trial decoded uncertainty. While one set contained trials where decoded uncertainty was greater on the previous trial than on the current trial, the other group contained trials where decoded uncertainty was greater on the current trial than on the previous trial. They reasoned that serial dependence effect should be greater on the second scenario. The results of van Bergen and Jehee (2019) agree with this prediction: reported orientation was more biased toward previously shown gratings when current trial decoded uncertainty was lower than decoded uncertainty of previous trial gratings. This provides evidence that serial dependence relies on an uncertainty based weighting of information, that this weighting relies on the probabilistic representation by a population of neurons (Pouget et al., 2013), and that decoded uncertainty from visual cortex at the time of stimulus encoding is used by downstream areas in the perceptual decision-making process.

In this commentary, I highlight two points implied by the main findings of van Bergen and Jehee's article: (1) that uncertainty from the encoded stimulus is stored in short-term memory and (2) that knowledge about different statistical environmental regularities might affect how the brain integrate past information with current decisions. While the first should inform the development of models of short-term memory storage, specially those concerning short-term synaptic plasticity, a candidate mechanism to explain serial dependence (Barbosa et al., 2020), the second has important implications for how we understand the accumulation of prior information and how it affects current behavior.

For downstream areas to have access to previous trial probability distribution of stimulus values, the brain must keep it in short-term memory. van Bergen and Jehee's (2019) results imply that the brain is able to maintain a representation of uncertainty in short-term memory. Although some computational models of short-term memory account for the maintenance of stimulus uncertainty throughout a delay period (Ma et al., 2014), probability population coding models are based on the assumption that the memoranda is kept through persistent activity of the population of neurons (Pouget et al., 2013). Serial dependence, on the other hand, might not rely on persistent spiking activity. Recent studies have shown that, differently than explicit short-term memory tasks, previous trial information cannot be decoded from EEG (Barbosa et al., 2020; Fornaciai and Park, 2020) and spiking activity (Barbosa et al., 2020) for most of the inter-trial interval. These results suggest that serial dependence relies on short-term synaptic plasticity to keep information from one trial to the next (Mongillo et al., 2008). However, one must be cautious to generalize results of activity-silent mechanism from studies that both measure and model neuronal activity in prefrontal cortex (e.g., Barbosa et al., 2020) to every task and context, as different regions of the brain might store information during the inter-trial interval through different mechanisms. Future studies should characterize the activity of different regions that might store information in short-term memory. In addition, future probability population coding models should account for uncertainty representation through short-term synaptic plasticity, either as a distributed representation of synaptic weights in the network or as a summary statistics extracted from the population activity and later used in the integration of previous and current trial information.

If serial dependence relied only on averaging of previous and current information, one would expect response biases to increase as the difference between consecutive stimuli increase. However, as first shown by Fischer and Whitney (2014), van Bergen and Jehee's (2019) results show a specific pattern of responses where serial dependence peaks for small differences between consecutive stimuli. The authors propose that such de-weighting for dissimilar stimuli emerges because the brain exploits the temporal stability of the environment (Dong and Atick, 1995). The model that better explained participants' behavior explicitly included an internal model of natural temporal statistics of visual orientation and outperformed a model misinformed about such temporal dynamics. van Bergen and Jehee (2019) suggest that participants use knowledge about the temporal dynamics of natural stimuli combined with previous sensory information to predict the upcoming stimulus (for similar results and theoretical proposal, see Kwon and Knill, 2013; Cicchini et al., 2018). It is important to note that the non-linear serial dependence pattern commonly observed in orientation perception studies is not ubiquitous in the literature, as previous studies have shown a linear relationship between current trial performance and the difference between current and previous trial stimuli (e.g., Cicchini et al., 2014). This difference could be attributed to a number of factors, including differences in stimulus processing, contextual differences, presence or absence of an internal model of environmental dynamics to the observer, or even the range of stimuli used in the experiment, which could be well-approximated by a linear model (e.g., Cicchini et al., 2018). The issue of why serial dependence appears to be non-linear for some studies and linear for others should be addressed by future studies.

Interesting predictions for future investigations of serial dependence can be generated by assuming that participants have at least an approximate knowledge of the natural temporal dynamics of the environment. For example, by having participants learn a new internal model of stimuli sequence (Chalk et al., 2010), serial dependence effect should change accordingly. However, the specific pattern of errors that characterize the serial dependence curve can be explained without assuming the brain knows the temporal dynamics of the environment (Kalm and Norris, 2018). Kalm and Norris (2018) were able to explain such pattern by assuming that past information was a mixture of decaying posterior distributions of previously shown stimuli. They suggest that serial dependence is a consequence of how past experiences are kept in memory and integrated with incoming sensory information. To solve this conundrum, these different models should be confronted in an independent study with an experimental design that allows to maximally differentiating them (Palminteri et al., 2017). As an initial but coarse suggestion, comparing participants' performance in two conditions with clearly different statistical dependencies across stimuli might be able to arbitrate between model predictions. Whereas, van Bergen and Jehee (2019) model would predict that serial dependence would differ across contexts with different statistical regularities, Kalm and Norris (2018) model would predict no difference in serial dependence effect as long as local statistics are kept the same.

Evidence that the brain uses past information and weight current and previous information based on uncertainty has been out there for a while. van Bergen and Jehee's (2019) work provide evidence in favor of theories that postulate that the brain processes information using probability distributions across stimuli values, and that the decision-making process at downstream areas is affected by the encoded and stored sensory uncertainty. Furthermore, it suggests that serial dependence emerges as a consequence of information weighting based on stimulus uncertainty and the stability of natural environments. Finally, this study opens new possibilities for studying the mechanism behind serial dependence and short-term memory.

## Author Contributions

The author confirms being the sole contributor of this work and has approved it for publication.

## Conflict of Interest

The author declares that the research was conducted in the absence of any commercial or financial relationships that could be construed as a potential conflict of interest.
